# When phenology matters: age–size truncation alters population response to trophic mismatch

**DOI:** 10.1098/rspb.2014.0938

**Published:** 2014-10-22

**Authors:** Jan Ohlberger, Stephen J. Thackeray, Ian J. Winfield, Stephen C. Maberly, L. Asbjørn Vøllestad

**Affiliations:** 1 Centre for Ecological and Evolutionary Synthesis, Department of Biosciences, University of Oslo, PO Box 1066 Blindern, 0316 Oslo, Norway; 2 Lake Ecosystems Group, Centre for Ecology & Hydrology, Lancaster Environment Centre, Library Avenue, Bailrigg, Lancaster, Lancashire LA1 4AP, UK

**Keywords:** age–size truncation, climate change, density dependence, phenological mismatch, population variability

## Abstract

Climate-induced shifts in the timing of life-history events are a worldwide phenomenon, and these shifts can de-synchronize species interactions such as predator–prey relationships. In order to understand the ecological implications of altered seasonality, we need to consider how shifts in phenology interact with other agents of environmental change such as exploitation and disease spread, which commonly act to erode the demographic structure of wild populations. Using long-term observational data on the phenology and dynamics of a model predator–prey system (fish and zooplankton in Windermere, UK), we show that age–size truncation of the predator population alters the consequences of phenological mismatch for offspring survival and population abundance. Specifically, age–size truncation reduces intraspecific density regulation due to competition and cannibalism, and thereby amplifies the population sensitivity to climate-induced predator–prey asynchrony, which increases variability in predator abundance. High population variability poses major ecological and economic challenges as it can diminish sustainable harvest rates and increase the risk of population collapse. Our results stress the importance of maintaining within-population age–size diversity in order to buffer populations against phenological asynchrony, and highlight the need to consider interactive effects of environmental impacts if we are to understand and project complex ecological outcomes.

## 1. Introduction

Phenological shifts (i.e. changes in the timing of periodic life-history events such as reproduction) are among the best-documented ecological impacts of climate change [1–3]. These shifts may arise through micro-evolutionary processes or represent phenotypic plasticity in traits affecting phenology [4]. Because species within the same food web may differ in the magnitude of their responses to climate change, phenological shifts have the potential to cause temporal mismatch between interacting species such as predators and their prey [5,6]. It has been shown recently that strong intrinsic density regulation (e.g. owing to competition) can buffer population growth against phenological mismatch [7]. This suggests that the demographic structure of a population, which determines the type and strength of intraspecific interactions, mediates how shifts in phenology and trophic interactions translate into changes in population abundance. Therefore, truncation of the population age–size structure, as commonly caused by exploitation [8,9] or disease outbreaks, may alter the population response to phenological shifts associated with climate change.

**Figure 1.**
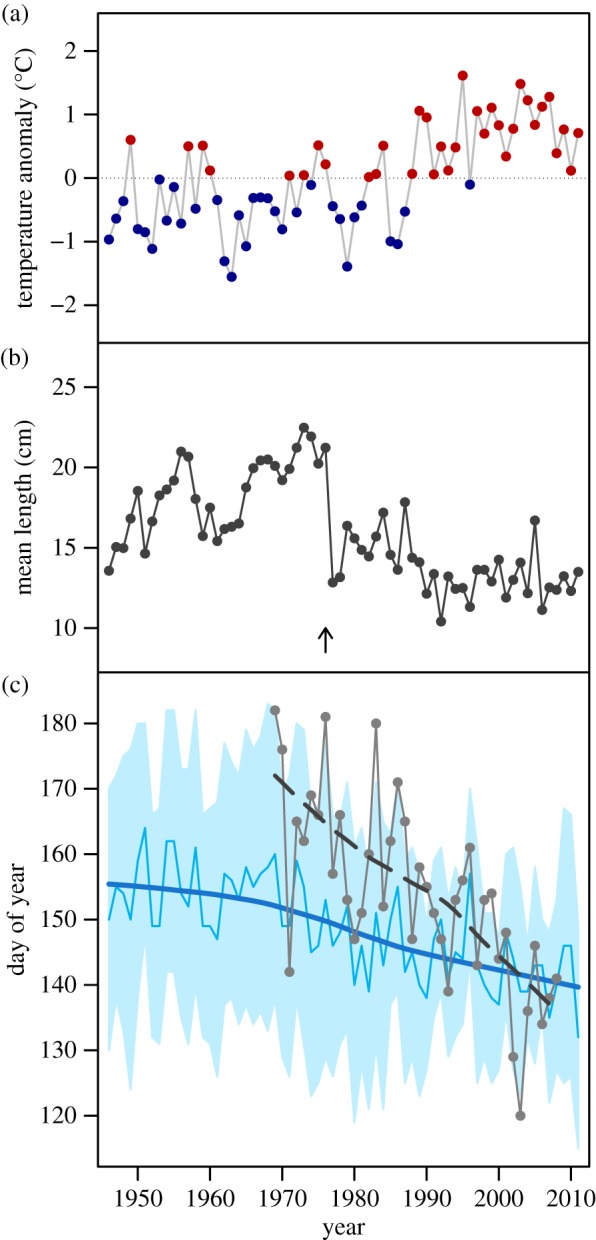
(*a*) Time series of annual temperature anomaly, (*b*) mean size of perch spawners and (*c*) predator (perch larvae, blue) and prey (zooplankton, grey) phenology. (*a*) Temperatures in Windermere have increased considerably since the late 1980s (above average values in red). (*b*) The mean sizes were severely reduced due to a disease outbreak in 1976 (arrow). (*c*) The timing of hatching of perch larvae is shown as duration (blue bands), peak (thin blue line) and long-term trend (thick blue line). Note the shorter larval hatching periods towards the end of the time series. The timing of zooplankton spring population development is shown as peak (grey circles and line) and long-term trend (thick dashed grey line). (Online version in colour.)

We test the hypothesis that the demographic structure of a population determines its sensitivity to phenological mismatch using long-term data on the phenology and dynamics of a freshwater fish, European perch (*Perca fluviatilis*, 67 years), and its zooplankton prey (Cladocera, 40 years) in Windermere, UK. Perch larvae rely on zooplankton as their primary food source upon depletion of their yolk reserves shortly after hatching in late spring, and are subsequently subjected to intraspecific competition and cannibalism by older perch [10]. Owing to a significant warming of the lake in recent decades (figure 1*a*), and a severe age–size truncation of the perch population in response to a disease outbreak in 1976 [11] (figure 1*b*), this long-term monitoring provides a unique opportunity to simultaneously study consequences of climatic and demographic change in a predator–prey system. Specifically, two mechanisms may contribute to higher population sensitivity to phenological shifts. First, a broad age–size distribution may result in a longer reproductive period, and therefore buffer populations against variability in prey phenology by reducing the probability of severe mismatch. Second, age–size truncation can lead to relaxed intrinsic density dependence if older individuals cannibalize or compete with young recruits, which may weaken the top-down control of recruitment and increase population sensitivity to temporal mismatch between predator larvae and their prey.

## 2. Material and methods

### (a) Data

Data are analysed from Windermere in the English Lake District, UK, one of the most intensively studied lakes in the world [12]. The scientific sampling of perch started in the 1940s and continues to date with very little change in gear type and fishing methods. Perch are caught on the spawning grounds for at least six weeks in spring (April–June) with traps that are unselective for individuals of 90–300 mm total length [13]. The traps are retrieved once a week and the total length of each individual is measured [14]. We used data on the time and size at capture of mature perch (classified with respect to spawning as either ‘ripe’ or ‘spent’ upon examination) from the Green Tuft spawning site in the North Basin of Windermere from the years 1946 to 2012. The Windermere perch population experienced a major disease (pathogen) outbreak in 1976, which induced a massive mortality, mostly among large mature individuals [11], and dramatically truncated the demographic structure of the population for many years [12,15]. Surface water temperature in Windermere, which has warmed considerably since the late 1980s, was recorded as part of the long-term monitoring at near daily intervals. Zooplankton are an important component of the diet of perch larvae, with *Daphnia* constituting the most abundant of all food organisms consumed by young perch [16]. Zooplankton abundance was derived from counts of Cladocera on filter papers used to estimate phytoplankton chlorophyll *a* [17]. The analysis of the phenological match/mismatch between perch larvae and zooplankton was performed for the period 1969–2008, during which consistent methods were used to collect all data at weekly to fortnightly intervals.

### (b) Peak and duration of perch spawning

We estimated the peak and duration of the spawning period for each year (1946–2012) by fitting normal distributions to the weekly catches of spawners (electronic supplementary material, figure S1). We used the mean of the distribution to estimate the peak spawning and four times the standard deviation (±2 s.d.) in order to estimate the duration of the spawning period (time during which approx. 95% of fish spawn). Multiple linear regressions were performed to independently model the peak and duration of the spawning season, with mean body size and the total number of mature fish caught as biotic predictors, and monthly/seasonal mean temperatures and the disease outbreak as abiotic predictors (centred variables). We allowed for quadratic temperature effects and tested for an interaction between temperature and the presence/absence of the disease. We selected the best temperature predictor, constructed the full model using this temperature measure and all other predictors, and iteratively dropped predictors/interactions from the model using leave-one-year-out cross-validation (see below).

### (c) Perch larvae–zooplankton match/mismatch

We estimated the time of hatching of perch larvae based on the temporal spawning distribution and the water temperature during subsequent egg development. According to experimental results, the heat sum in degree-days required for perch larvae to hatch is constant over a wide range of temperatures, including those experienced by perch larvae in Windermere [18]. We computed the number of days required for the larvae to hatch based on the temperature experienced in a given year and a heat sum of 97 degree-days above a threshold of 4.9°C [18]. Peak spawning in Windermere perch typically occurs at water temperatures around 12°C. At these temperatures, approximately 80% of larvae hatch within a period of 1–2 days approximately two weeks after spawning [18]. Using phenology measures for zooplankton spring population development [17], which is consistent with a normal distribution over time [19], we calculated the phenological match/mismatch as the difference in days between the estimated peaks of larval hatching and zooplankton abundance (electronic supplementary material, figure S2).

### (d) Perch recruitment

Recruitment was taken as the abundance of 2-year-old perch (the youngest age-class caught in the trap survey) from recent estimates of age-specific abundances until 2002 [20]. Multiple linear regressions were performed to model recruitment (log-transformed number of recruits) using the following predictors: the duration of the spawning/hatching period, the annual match/mismatch index, the number of spawners and average winter temperature (allowing for quadratic effects of the match/mismatch index, temperature and the number of spawners). The number of age 3+ perch present during the year class's first year of life was used to test for potential effects of competition and/or cannibalism among age-classes, which are known to be important intraspecific processes in perch [10]. All continuous predictor variables were centred for the analysis. Interactions were tested for between the two phenological variables and the degree of competition/cannibalism experienced by young perch using a dummy variable (low/high) based on the median number of age 3+ perch. The dummy was used instead of the continuous predictor in order to avoid over-fitting of the model. The temperature predictor was selected based on the best predictive power of a model containing only one temperature measure as covariate. We then built the full model using this temperature measure and all predictors/interactions under scrutiny, and iteratively dropped terms from the model using leave-one-year-out cross-validation (see below).

### (e) Model selection and validation

Model selection was performed using backward selection by starting with a full model that contained all predictors and interactions under scrutiny. At each step, we performed leave-one-year-out generalized cross-validation by computing the square root of mean-squared out-of-sample prediction errors (leaving one data point out at a time). This approach provides a direct measure of the predictive power of a model [21] and helps to avoid over-fitting [22]. The cross-validation procedure involved the following steps: (i) fitting the model to the dataset with one year removed, (ii) predicting the observation not used when fitting the model, (iii) calculating the prediction error (predicted-observed), (iv) repeating the above procedure for all years, and (v) calculating the square root of the mean-squared prediction errors. Once the optimal model was selected by minimizing the cross-validation criterion, we validated the model by testing for autocorrelation, homogeneity and normality of residuals. In addition, we performed an automated model selection based on AICc, which confirmed the results obtained using the leave-one-year-out cross-validation procedure (see the electronic supplementary material).

### (f) Coefficient of variation in abundance

In order to illustrate changes in population variability, the coefficient of variation of the perch abundance time series was calculated for the two periods before and after the main age–size truncation caused by the disease outbreak in 1976. The coefficient of variation was computed as the ratio of the standard deviation to the mean for a sliding window of 3–11 years.

All statistical analyses were performed in R (v. 3.0.2 [23]).

## 3. Results

Since the 1940s, the spawning period of perch (and thus the hatching of perch larvae) has advanced by about two weeks and shortened by about one week (figure 1*c*). This shift in reproductive timing towards earlier and shorter spawning seasons is associated with changes in climate and population size structure (electronic supplementary material, figure S3). The linear model of peak spawning (PS) included the number of spawners (*S*), the disease outbreak (*P*) as factor and linear/quadratic terms of spring temperature (ST): 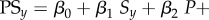 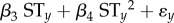, where *β*s are regression coefficients and *ɛ_y_* is an error term (electronic supplementary material, table S1). The linear model of the length/duration of the spawning period (LS) included the mean size of spawners (MS), the number of spawners (*S*) and linear and quadratic terms of lake temperature in May (MT): 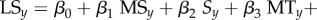 
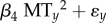
, where *β*s are regression coefficients and *ɛ_y_* is an error term (electronic supplementary material, table S2). Consequently, both the peak and duration of the spawning season are associated with warmer STs and the disease-induced size truncation of the perch that caused a severe reduction in the mean body size of spawners. In line with these results, a linear mixed-effects model showed that the timing of spawning of individual fish is best explained by changes in temperature, whereas the variance (i.e. the period over which all individuals spawn in a given year) is best explained by the mean size (see the electronic supplementary material).

**Figure 2.**
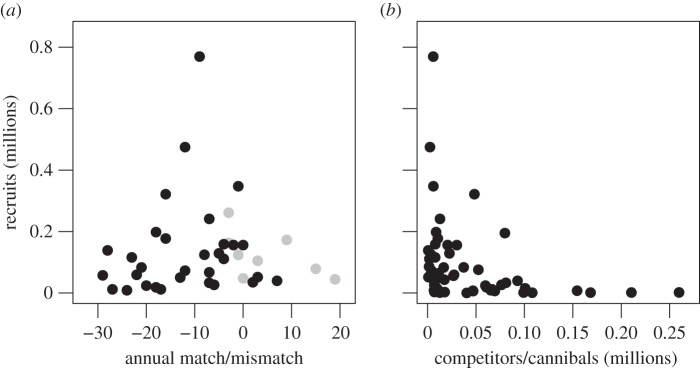
Number of recruits at age 2 as a function of (*a*) the match/mismatch 2 years earlier and (*b*) the number of age 3+ competitors/cannibals the previous year. (*a*) The annual match/mismatch index is the difference between peaks in larval hatching and zooplankton abundance. Perch recruitment was estimated for the years 2003–2010 (grey; see the electronic supplementary material). (*b*) Perch recruitment for all years with available age-specific data as a function of the number of older competitors/cannibals.

The phenology of zooplankton spring population development advanced even more rapidly than the spawning period of perch (1969–2008; figure 1*c*), in response to warmer water in spring and earlier growth of their phytoplankton food (for details, see [17]). As a consequence, the time difference between the peak hatching of perch larvae and the peak zooplankton abundance (i.e. the annual match/mismatch) has shifted considerably. During the 1970s, peak zooplankton abundance regularly occurred three to four weeks after the peak hatching of perch larvae, whereas during the most recent decade these two phenological events have consistently occurred less than one week apart (electronic supplementary material, figure S2). The relationship between the number of perch recruits at age 2 and the match/mismatch index illustrates that the relative timing of phenological events affects recruitment in perch (figure 2*a*). While low recruitment occurs at any given match/mismatch, highest recruitment occurs when peak hatching of larvae occurs approximately 10 days before the zooplankton peak.

**Figure 3.**
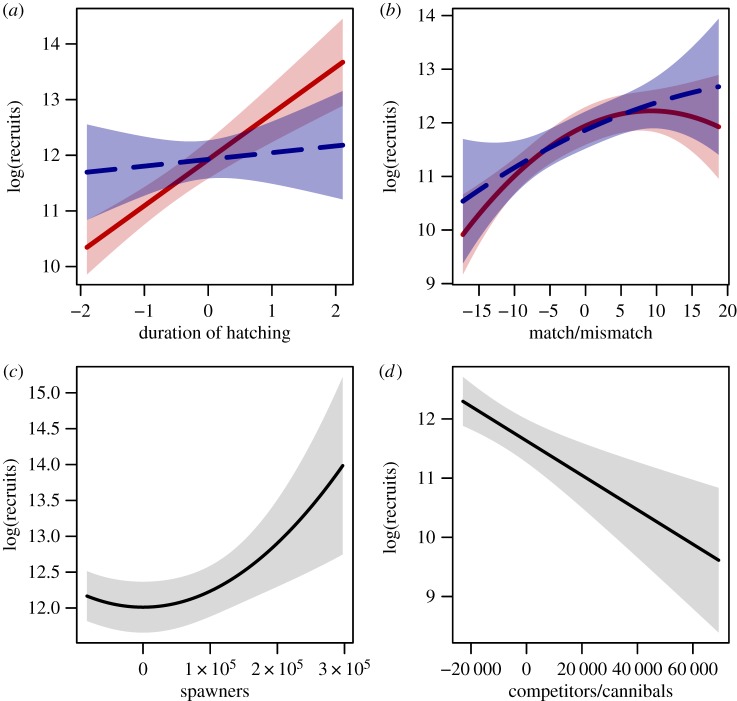
Effects of the selected multiple linear regression model for perch recruitment. Recruitment depends on the interactions between the level of competition/cannibalism (‘low’, red bands and solid line; ‘high’, blue bands and dashed line) and both (*a*) the duration of the larval hatching period and (*b*) the annual match/mismatch. Recruitment also depends on (*c*) the number of spawners and (*d*) the number of competitors/cannibals. Lines represent model predictions and the shaded areas 95% CIs. The non-significant temperature effect is not shown. (Online version in colour.)

**Figure 4.**
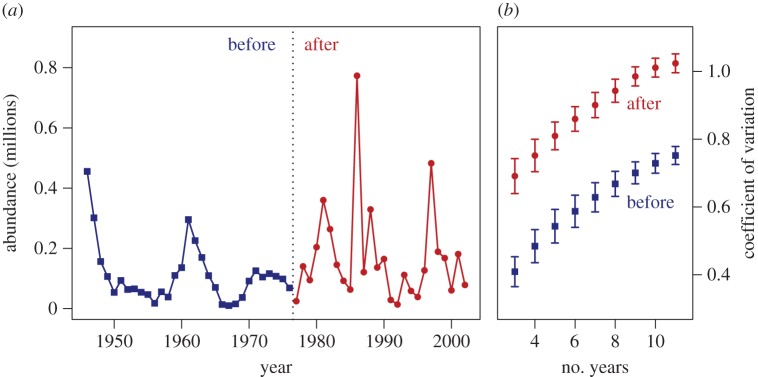
(*a*) Total population abundance over time and (*b*) coefficient of variation. The coefficient of variation was calculated for the two time periods before (blue line and squares) and after (red line and circles) the major age–size truncation caused by the disease outbreak in 1976, and is shown as a function of the number of years used in the sliding window approach (points represent means and bars standard errors of the means). (Online version in colour.)

The recruitment model for perch provides an explanation for this relationship. In addition to the number of spawners (*S_y_*), winter temperature (WT*_y_*_+1_) and the number of age 3+ perch (CA*_y_*_+1_, potential competitors/cannibals) in the first year of life of the year class, the selected model includes interactions between the degree of competition/cannibalism (*D*) and both phenology measures—the duration of the larval hatching period (LS*_y_*) and the match/mismatch index (PM*_y_*): 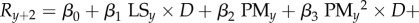 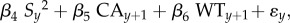 where *β*s are regression coefficients and *ɛ_y_* is an error term. As expected, recruitment increases with the number of spawners (above a certain threshold value), but decreases with the number of competitors/cannibals (figure 3). The positive effects of the duration of the larval hatching period and the quadratic effect of the annual match/mismatch are significant at low (but not significant at high) competition/cannibalism. The model thus reveals that perch recruitment only depends on the relative timing of phenological events when competition/predation within the population is weak (table 1). In other words, a relaxing of intrinsic density regulation increases the population sensitivity to the timing of phenological events. The degree of competition/cannibalism in interaction with the phenology variables explains most of the variance in the perch recruitment time series. Dropping the interactions and the main effect of competition/cannibalism, while keeping all other predictors, decreases the variance explained by the model from 82 to 29%. Furthermore, dropping the phenology variables and their interactions, while keeping the main effect of competition/cannibalism, leads to a similar drop in variance explained from 82 to 38%. The high recruitment variance at low levels of density regulation is also illustrated by the relationship between the number of recruits and the number of potential competitors/cannibals during the recruits first year of life (figure 2*b*). High recruitment variance translates into elevated variability in total abundance, because the number of older fish is reduced and recruits dominate the population. Accordingly, the perch abundance time series exhibits a clear increase in population variability associated with the disease-induced age–size truncation (figure 4). Consequently, the effect of the trophic mismatch on overall perch abundance strongly depends on the demographic structure within the population.

**Table 1.** Results of the multiple linear regression model of perch recruitment. Predictors: degree of competition/cannibalism (*D*), length of larval hatching period (LS*_y_*), predator–prey match/mismatch index (PM*_y_*), number of spawners (*S_y_*), number of competitors/cannibals in the first year of life (CA*_y_*_+1_), WT in the first year of life (WT*_y_*_+1_). Significance levels of *p*-values are denoted by asterisks.

coefficient	effect	estimate	s.e.	*p*-value
*β*_0_		11.4828	0.1709	<0.0001***
*β*_1_	LS*_y_* × *D*^low^	0.8295	0.1312	<0.0001***
	LS*_y_* × *D*^high^	0.1209	0.2038	0.5593
*β*_2_	PM*_y_*	0.0607	0.0116	<0.0001***
*β*_3_	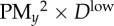	−0.0033	0.0014	0.0239*
	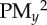	−0.0009	0.0018	0.5987
*β*_4_	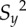	2.2 × 10^−11^	6.9 × 10^−12^	0.0038**
*β*_5_	CA*_y_*_+1_	−2.9 × 10^−5^	7.3 × 10^−6^	0.0007***
*β*_6_	WT*_y_*_+1_	−0.1637	0.1009	0.1198

***p < 0.001, **p < 0.01, *p < 0.05.

## 4. Discussion

Our analyses demonstrate that (i) the age–size structure of a population, in addition to climate, affects the timing of reproduction and trophic interactions, and (ii) age–size truncation increases population sensitivity to phenological mismatch and ultimately elevates variability in population abundance by relaxing intraspecific density dependence (i.e. the intrinsic top-down control of recruitment). The study thereby contributes to our understanding under which conditions phenological mismatch is likely to be important, and how climate-induced shifts in phenology may interact with other agents of environmental change such as disease spread or exploitation.

Most populations in seasonal environments have distinct reproductive periods that are temporally linked to the phenology of resources and predators [24,25] as a result of past selection pressures and shared environmental cues. Temporal mismatch between predator reproduction and the timing of optimal food supply can decrease population growth of the predator [26]. In fish populations, the period following larval hatching is thought to be critical for offspring survival, because larvae must quickly find suitable prey upon depletion of their yolk reserves [24]. Spawning typically occurs over several weeks, thereby ensuring that at least a small proportion of offspring in each year encounter sufficient food to survive. A long reproductive period potentially reduces interannual variance in offspring survival [27], as it buffers impacts of environmental fluctuations such as climate-dependent variability in prey abundance. Accordingly, it has been suggested that the duration of the spawning period can have a substantial effect on recruitment variability [28]. Here, we demonstrate that such a risk-spreading strategy can be undermined by age–size truncation, if the timing and/or duration of the reproductive period depend on individual body size. Previous studies have shown that larger, older fish tend to arrive earlier at spawning sites than first-time spawners, and that the spawning duration of young age-classes can be only half that of older individuals [27]. We find that the mean body size of spawners increases over the reproductive period (electronic supplementary material, figure S4), suggesting that large individuals mature over extended periods of time and/or spawn later when compared with small individuals. A reduction in mean spawner size thus causes shorter reproductive periods (electronic supplementary material, figure S3). This demographic shift in perch reflects direct and indirect effects of the disease outbreak, which caused a selective removal of larger fish and induced a phenotypic shift towards a smaller size at maturation, both of which lead to a decrease in the mean size [15]. Analogous changes can be expected in response to size-selective exploitation such as in fisheries, which reduces the mean size of a population [27,29], and has been shown to induce phenotypic shifts in size at maturation [30,31].

In addition to its direct effect on reproductive timing, demographic structure affects offspring survival after the larval stage via density-dependent processes, and thus determines how post-larval abundance translates into adult abundance. Populations with strong intraspecific competition are buffered against phenological mismatch [7]. Moreover, it is known that exploited fish populations show higher variability and short-term fluctuations in abundance compared with unexploited populations [9,29,32]. It has been suggested that the link between age–size truncation and variability in abundance may be explained by increased population sensitivity to stochastic environmental impacts or changes in demographic parameters such as density regulation [9]. Our results demonstrate that changes in density dependence (i.e. reduced intraspecific cannibalism/competition) due to age–size truncation can cause elevated population variability. Our recruitment model further suggests that, once the population age–size structure is truncated and density dependence is relaxed, population variability is mainly driven by changes in phenology rather than environmental stochasticity in general. Relaxed density regulation accentuates the effect of phenological asynchrony on recruitment, because it otherwise reduces survival after the trophic (larvae–zooplankton) interaction. Hence, while intrinsic dynamics like cyclic behaviour (e.g. due to cohort resonance effects) are unlikely to be the ultimate cause of temporal fluctuations in natural populations [33], the lack of intrinsic density regulation reveals the impact of extrinsic impacts (e.g. prey phenology) on population abundance. Increased population variability poses major ecological and economic challenges as it can diminish sustainable harvest rates and increase the risk of population collapse [29,34]. Our findings thus stress the importance of maintaining within-population age–size diversity as it can buffer populations against phenological asynchrony associated with climate change.

Finally, if the timing of reproduction is a partly heritable trait [35], our finding that recruitment was weakly linked to the phenological timing before the major shift in population size structure implies that selection for optimal spawning date may have been weak prior to the demographic truncation. Our results thus suggest that age–size truncation may increase selection pressures on traits affecting population phenology, because offspring survival is more tightly linked to phenological events when the demographic structure has been eroded.

## Supplementary Material

Electronic Supplementary Material (ESM)

## Data accessibility

Data used in this study are available at the NERC-Environmental Information Data Centre (doi:10.5285/453fdf49-7328-42ec-94b7-1cebf06c51e2; doi:10.5285/495fd597-42b2-4985-96dc-3d9ec024b829; doi:10.5285/85dc93d2-8c29-4660-b725-793e43703e46).
