# Supplementary material for: When phenology matters: age–size truncation alters population response to trophic mismatch
Source: Proc Biol Sci. 2014 Oct 22;281(1793):20140938. doi: 10.1098/rspb.2014.0938 (PMC4173671; doi:10.1098/rspb.2014.0938)
Supplement: Electronic Supplementary Material (ESM) [file rspb20140938supp1.pdf]

ELECTRONIC SUPPLEMENTARY MATERIAL (ESM)

Perch recruitment in recent years: Since data on perch recruitment were only available up to 2002, we estimated recruitment in recent years using the following correlation approach: we estimated the slope and intercept of the relationship between perch recruitment and the total catch of each year in our dataset for the years 1990-2002 (correlation=0.52) and used these estimates to predict recruitment based on the catch data for all years after 2002. We thereby assumed that recruits dominated the catch in the years after 1990. These estimates for recent years are shown on Fig. 2, but were not used in the recruitment model.

Model selection: We performed an alternative model selection for the recruitment model based on AICc using the *dredge* function from the package 'MuMIn' (v.1.10.0 [1]) in R (v.3.0.3 [2]). The two best models selected by this procedure had a delta AICc of < 2 and are in line with the results from the leave-one-year-out cross validation procedure. One of the two 'best' models included the same main effects and interactions as presented in Table 1, while the other model only dropped the (non-significant) temperature term.

Perch recruitment model outlier test: We computed Bonferroni p-values for the Studentized residuals to test for outliers in the linear recruitment model. Based on this test the recruitment year 1997 was removed from the analysis (p=0.042), which increased the variance explained, but did not affect the significance levels of the estimated parameters.

Sensitivity test: We tested the sensitivity of our results to the estimation of the peak and duration of the perch spawning season by excluding data from years in which the highest weekly catches had been recorded in the 1st week of sampling, since the peak of the spawning season was possibly missed in those years (1961, 1980, 1981, 1982, 1988, 1990, 2003). Using the reduced dataset yielded the same model selection and qualitative results (approximate significance levels of parameters and deviance explained by the models).

Linear mixed effects model of individual spawning time: We used linear mixed effects models (LME) to model changes in the timing of spawning based on individual observations of mature perch on the spawning grounds (N=63,093). Random effects accounted for the lack of independence of the data within years. The model was constructed starting with the full model including all explanatory variables and factors. We tested for random effects by comparing the linear model to the same model including random intercepts using a likelihood ratio test based on a chi-squared distribution (restricted maximum likelihood parameter estimation, REML). We compared different variance structures for the full model and selected the best variance structure based on a likelihood ratio test. We then excluded non-significant fixed effects from the model, one at a time, based on p-values (maximum likelihood parameter estimation, ML). Model selection based on the AIC yielded results that were consistent with the likelihood ratio test. The LME was fit using the package 'nlme' (v.3.1-102 [3]) in R (v.3.0.3 [2]). The selected model for the timing of spawning of individual perch included spring temperature (SpT), the pathogen presence/absence (P), and an interaction between body size (BS) and sex/gender (G): $WS_{i,y} = \beta_0 + \beta_1 SpT + \beta_2 P + \beta_3 BS + \beta_4 G + \beta_5 BS \times G + b_y + \epsilon_{i,y}$, where $WS_{i,y}$ is the week of the year an individual (i) was caught on the spawning ground, βs are coefficients of the fixed effects, b_y is the normally distributed ($\sim N(0, \sigma_b^2)$) random year group effect, and $\epsilon_{i,y}$ is a normally distributed error term ($\sim N(0, \sigma^2)$). The variance in residuals ($\epsilon_{i,y}$) was modeled as a power function of the mean size of all individuals of that year: $var(\epsilon_{i,y}) = \sigma^2 \times |BS_{i,y}|^{2\delta}$. The linear mixed effects model thus showed that the timing of spawning for an individual fish is most strongly affected by temperature. The mean size was retained in the model as fixed effect, but the overall effect size was small. Temperature thus explained most of the inter-individual differences in spawning timing. The variance structure was best explained by the annual mean size, suggesting that the duration of the spawning period was most strongly affected by the mean size of the spawners in a given year.

REFERENCES

1. Bartoń, K. 2014 MuMIn: Multi-model inference. R package version 1.10.0. http://CRAN.R-project.org/package=MuMIn.

2. R Development Core Team 2013 *R: A language and environment for statistical computing*. Vienna, Austria: R Foundation for Statistical Computing.

3. Pinheiro, J. & Bates, D. 2010 *Mixed-Effects Models in S and S-PLUS*. Springer.

TABLES

Table S1: Results of the multiple linear regression model of peak spawning in perch. The model included the effects of the number of spawners (S_y), the pathogen (P), and spring temperature (ST_y).

Coefficient	Effect	Estimate	SE	P-value
β_0		20.2556	0.1179	<0.0001 ***
β_1	S_y	-0.0002	0.0001	0.0391 *
β_2	P	-1.5198	0.1651	<0.0001 ***
β_3	ST_y	-0.4404	0.0731	<0.0001 ***
β_4	${ST_y}^2$	-0.1114	0.0484	0.0249 *

Significance levels: '***' <0.001, '**' <0.01, '*' <0.05, '.' <0.1

Table S2: Results of the multiple linear regression model of the duration of perch spawning. The model included the effects of the mean size of spawners (MS_y), the number of spawners (S_y) and May temperature (MT_y).

**Coefficient**	**Effect**	**Estimate**	**SE**	**P-value**
β_0		5.2707	0.1189	<0.0001 ***
β_1	MS_y	0.0002	0.0001	0.0753 .
β_2	S_y	0.2346	0.0321	<0.0001 ***
β_3	MT_y	-0.2033	0.0725	0.0068 **
β_4	${MT_y}^2$	-0.1337	0.0436	0.0032 **

Significance levels: '***' <0.001, '**' <0.01, '*' <0.05, '.' <0.1

FIGURE LEGENDS

Figure S1: Annual catches by week (1946-2013) and fitted normal distributions. Red circles and bars at the bottom of each plot indicate the estimated peak and spawning duration.

Figure S2: Annual values and temporal trend in the match/mismatch index. The index was calculated as the time difference between peak hatching of perch larvae and the peak in zooplankton abundance in spring. The thick grey line was fitted using loess smoother with a smoothing parameter (span) of 2/3 to illustrate the temporal trend in the match/mismatch.

Figure S3: Multiple linear regression results of the peak (left) and duration (right) of perch spawning. Peak spawning timing was associated with the disease/pathogen outbreak (top), average spring temperature (centre), and the number of fish caught (bottom). Similarly, the duration of the spawning period was associated with the mean size of spawners (top), average temperature in May (centre), and the number of fish caught (bottom).

Figure S4: Body size of male (light grey) and female (dark grey) perch spawners by week for the years 1946-2012. Thick black lines represent median values, boxes represent the 1st and 3rd quartiles, and whiskers represent one interquartile range from the boxes. The width of the boxes indicates the number of data points per week and sex.

FIGURES

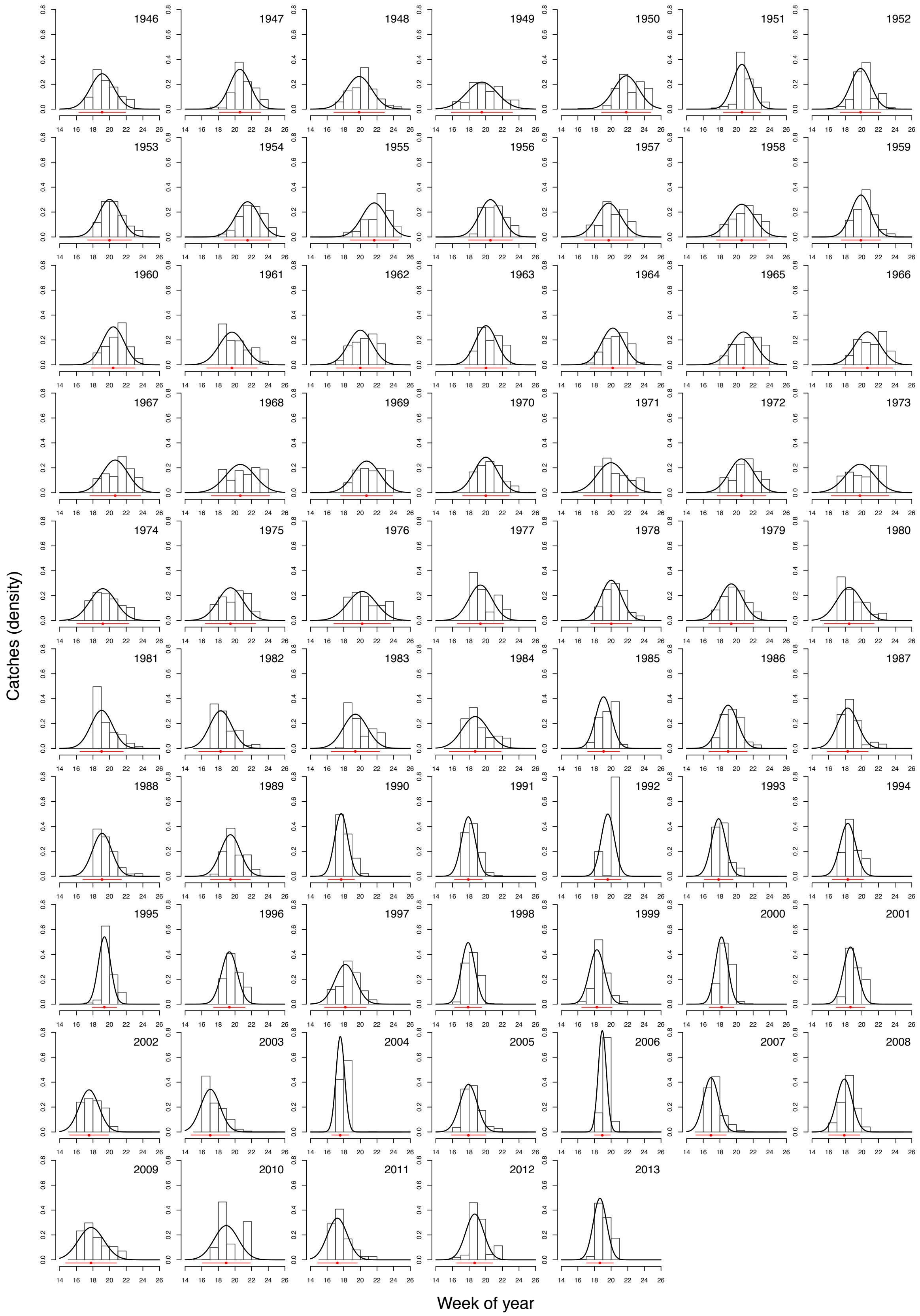

Fig. S1

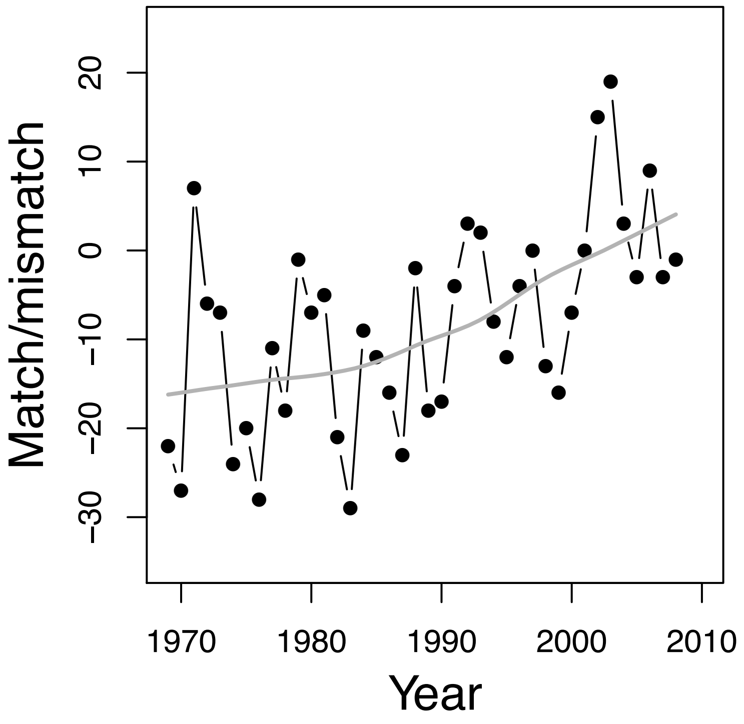

Fig. S2

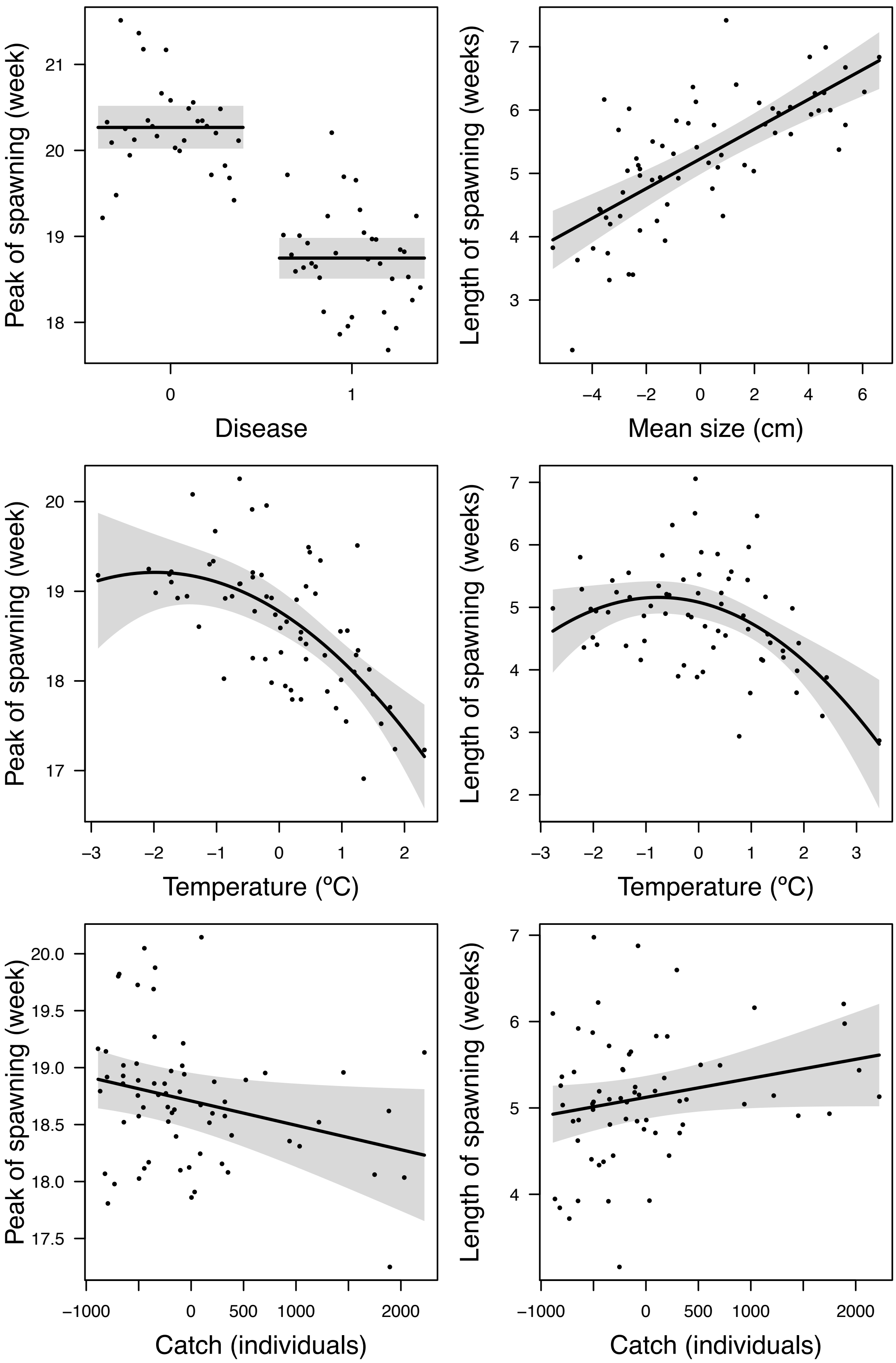

Peak of spawning (week)
Disease
Length of spawning (weeks)
Mean size (cm)
Peak of spawning (week)
Temperature (ºC)
Length of spawning (weeks)
Temperature (ºC)
Peak of spawning (week)
Catch (individuals)
Length of spawning (weeks)
Catch (individuals)

Fig. S3

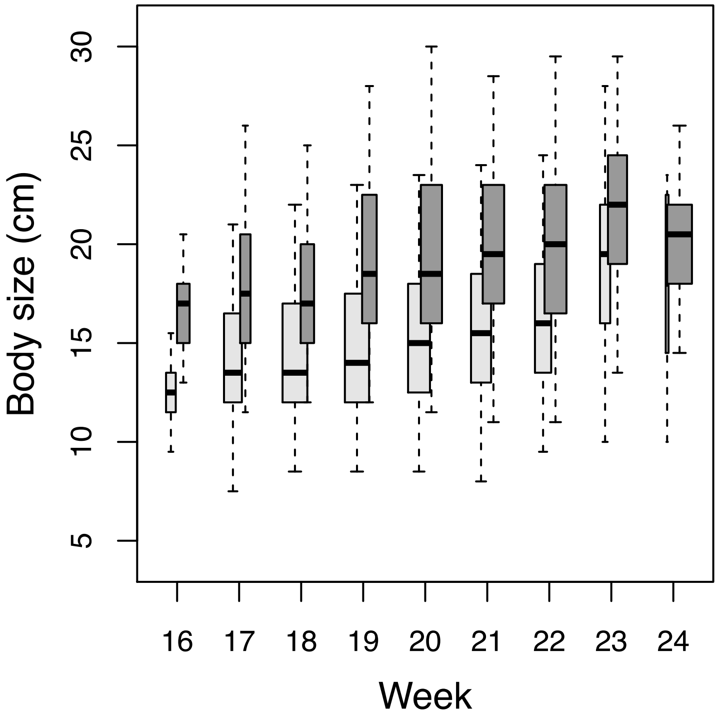

Fig. S4
